# Correlation study: Bone density and circulating inflammatory markers in postmenopausal patients

**DOI:** 10.1002/iid3.1365

**Published:** 2024-08-02

**Authors:** Xingyu Jin, Ye Wang, Huazheng Wang, Lu Wang, Binglong Huan, Chao Liu

**Affiliations:** ^1^ Department of Orthopedics, Suzhou BenQ Medical Center The Affiliated BenQ Hospital of Nanjing Medical University Suzhou China; ^2^ Department of Orthopedics Suzhou Industrial Park Xinghu Hospital Suzhou China

**Keywords:** blood routine, inflammatory index, postmenopausal osteoporosis, systemic immune‐inflammation index

## Abstract

**Objective:**

This study aims to investigate the correlation between changes in bone mineral density (BMD) in postmenopausal women and circulating inflammatory markers.

**Methods:**

This retrospective study focused on postmenopausal women admitted to the orthopedic department of Suzhou Benq Medical Center from June 2022 to December 2023, following predetermined inclusion and exclusion criteria. We retrospectively collected data on initial blood routine test results and bone density measurements for all study subjects upon admission, including parameters such as white blood cell count (WBC), C‐reactive protein, interleukin‐6 (IL‐6), and procalcitonin (PCT). Additionally, the systemic immune‐inflammation index (SII) was calculated using neutrophil count, lymphocyte count, and platelet count. Statistical analyses using SPSS and GraphPad software were performed to assess the correlation between bone density and inflammatory markers.

**Results:**

Patients were classified into three groups based on BMD results, including 60 individuals in the osteoporosis (OP) group, 127 individuals in the osteopenia group, and 37 individuals in the Normal group, respectively. Principal component analysis analysis suggested that WBC, SII, and postmenopausal OP (PMOP) held significant feature values. Correlation analysis indicated a correlation between WBC (*p* = 0.021), IL‐6 (*p* = 0.044), SII (*p* = 0.034), and PMOP. One‐way ANOVA analysis revealed significant differences in IL‐6 (*p* = 0.0179), SII (*p* = 0.0210), and PCT (*p* = 0.0200) among the three groups. Finally, ROC curve analysis demonstrated that SII (area under the curve = 0.716) has predictive value for PMOP.

**Conclusion:**

This study identified a certain predictive value for PMOP through the assessment of inflammatory markers in peripheral blood using routine blood tests.

## Graphical abstract

This study identified a certain predictive value for postmenopausal osteoporosis through the assessment of inflammatory markers in peripheral blood using routine blood tests.

## 1 INTRODUCTION

Postmenopausal osteoporosis (PMOP) is a chronic bone metabolism disorder characterized by bone loss, alterations in bone microstructure, and susceptibility to fragility fractures after menopause.^1^, ^2^ One possible reason is that estrogen promotes apoptosis of osteoclasts and inhibits their formation, while estrogen itself promotes osteogenic differentiation of mesenchymal stem cells. Therefore, the decline in estrogen levels after menopause may lead to disturbances in bone metabolism.^3^

With the advent of an aging population, PMOP has gradually emerged as a significant societal issue, imposing substantial health and economic burdens.^4^, ^5^ One of the most effective approaches to managing PMOP is its early diagnosis and treatment. Consequently, the focus has long been on the hotly debated research topic of how to efficiently and effectively screen for PMOP in its early stages.^5^

Research on the interplay between certain inflammatory markers and PMOP mechanisms has yielded insights, confirming a close association between systemic immunity, inflammation, and PMOP. For example, inflammatory conditions have been shown to adversely affect bone metabolism,^6^ and the renowned bone metabolism pathway, RANK‐RANKL‐OPG, has been demonstrated to be regulated by inflammatory factors.^7^ Additionally, studies indicate a correlation between an increased ratio of neutrophils to lymphocytes and decreased bone mineral density (BMD).^8^, ^9^ Studies have shown that some bacteria in the human body can produce some metabolites during its own metabolic process, and SCFAs are the main ones. In addition to the anti‐inflammatory effects, these metabolites may directly act on various types of osteocytes.^10^ Recent studies have also suggested a close interaction between immune system and bone metabolism, known as “osteoimmunology”, which represents the role of immune cells or immune related factors in modulating the bone metabolism.^11^

Complete blood count (CBC) provides readily accessible clinical information, encompassing various inflammatory markers such as white blood cell count (WBC), C‐reactive protein (CRP), procalcitonin (PCT), interleukin‐6 (IL‐6), and so on. The systemic immune‐inflammation index (SII) can be calculated using neutrophil count, lymphocyte count, and platelet count (SII = Neutrophil Count × Platelet Count/Lymphocyte Count).^12^ Hence, we aim to analyze the correlation between inflammatory markers in CBC and PMOP to explore the predictive value of these markers for PMOP. If CBC could serve as an initial screening tool for PMOP, it would hold significant implications for the early prevention and management of this condition.

## 2 DATA AND METHODS

### 2.1 Study population

Based on inclusion and exclusion criteria, a total of 224 postmenopausal female patients who visited the orthopedic department of Suzhou Benq Medical Center from June 2022 to December 2023 were retrospectively enrolled as the study population.

### 2.2 Inclusion criteria and exclusion criteria

Inclusion criteria: (1) patients older than 50 years with documented menopausal status; (2) patients diagnosed with postmenopausal osteoporosis (OP) according to the World Health Organization (WHO) criteria for OP diagnosis.^13^

Exclusion criteria: (1) patients with presence of primary immunodeficiency disorders, acquired immune deficiency, hematological disorders, psychiatric disorders, severe infections, or allogeneic blood transfusions within the past 3 months; (2) patients with malignancies, major organ failure, or organ transplants; (3) patients using immunomodulatory agents, glucocorticoids, or other hormone replacement therapy within the past year; (4) patients with other conditions affecting bone metabolism or immune regulation; (5) Patients taking medications affecting bone metabolism; (6) patients admitted to hospital due to serious trauma, such as car accidents and falling from a height.

### 2.3 Group division

Using dual‐energy X‐ray absorptiometry (DXA) to measure patients' BMD, and patients were categorized into three groups based on their lowest *T*‐scores: *T*‐score ≤ −2.5 were classified into the OP group; −2.5 < T‐score ≤ −1.0 were classified into the osteopenia (ON) group, and *T*‐score > −1.0 were classified into the normal (NO) group.

## 3 STATISTICAL ANALYSIS

**Figure 1:**
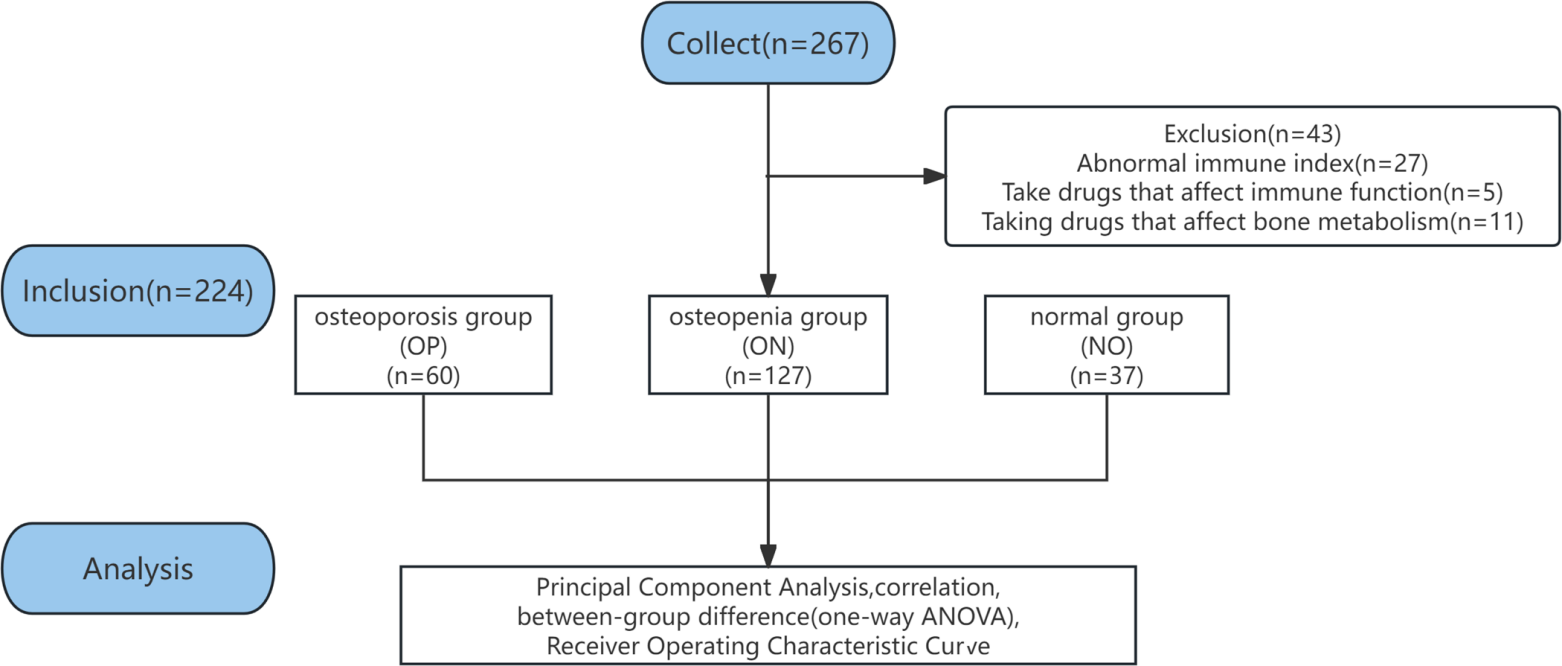
Flow chart.

Data analysis was conducted using GraphPad Prism version 9.3 (GraphPad Software) and SPSS version 26.0 (IBM Corp.). Continuous variables were presented as mean ± standard deviation (mean ± SD), while categorical variables were expressed numerically. Between‐group differences were analyzed using one‐way ANOVA. Principal component analysis (PCA) was employed to assess the impact of different variables on PMOP, with the selection of principal components of eigenvalues> 5. Pearson correlation analysis was utilized to examine data correlations. Receiver operating characteristic (ROC) curve analysis was performed to assess the predictive value of the criteria by calculating the area under the curve (AUC) and Youden index (Figure 1).

## 4 RESULTS

### 4.1 General characteristics

Based on the above inclusion and exclusion criteria, a total of 224 subjects with a mean age of 72.42 ± 11.61 years were enrolled. Based on the BMD results, participants were divided into three groups: the OP group comprised 60 subjects with a mean age of 76.24 ± 9.7 years, the ON group comprised 127 subjects with a mean age of 72.42 ± 11.46 years, and the NO group comprised 37 subjects with a mean age of 67.41 ± 12.48 years (Table 1).

**Table 1:** General information of the subjects.

	Total sample	Osteoporosis	Osteopenia	Normal	*p*
*N*	224	60	127	37	‐
Age, years	72.42 ± 11.61	76.04 ± 9.7	72.42 ± 11.46	67.41 ± 12.48	0.104
Height, m	1.56 ± 0.06	1.52 ± 0.04	1.58 ± 0.05	1.59 ± 0.05	0.014
Weight, kg	55.64 ± 10.34	52.00 ± 9.86	52.50 ± 8.17	64.65 ± 8.01	0.003
BMI	22.74 ± 3.75	22.41 ± 3.92	20.96 ± 3.07	25.60 ± 2.42	0.007

### 4.2 Principal component analysis

**Figure 2:**
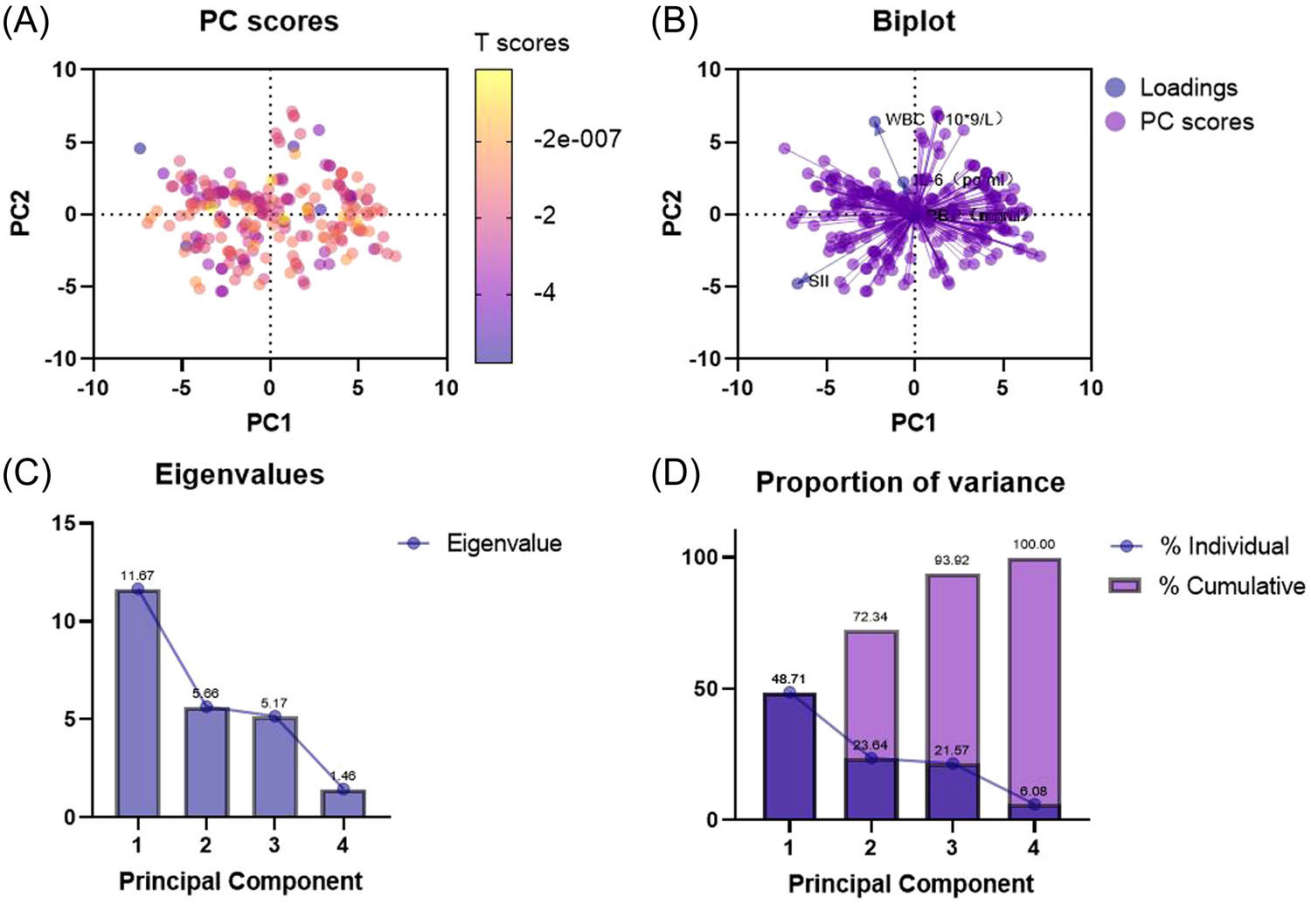
(A) Cluster differentiation among samples; (B) SII correlates with PC2, while WBC correlates with PC1; (C and D) Scree plot shows that the characteristic root value is large and the change is obvious, which makes a great contribution to explaining the change of bone mass. It can be seen that extracting the first two factors has a significant effect. SII, systemic immune‐inflammation index; WBC, white blood cell count.

PCA analysis was utilized to assess the impact of various measurements on the subjects, including WBC, IL‐6, SII, PCT, and CRP. Two potential PC values were selected when eigenvalues > 5: PC1 eigenvalue = 11.67 and PC2 eigenvalue = 5.662, together contributing to a cumulative variance of 72.34% (PC1: 48.71%, PC2: 23.64%, see Figure 1). The PCA results revealed that WBC and SII held significant feature values, with evident cluster differentiation in the data. The scree plot indicated that the first two factors contributed more significantly to the interpretation of the results (Figure 2).

### 4.3 Correlation between inflammatory markers and PMOP

To explore the association between inflammatory markers in routine blood tests and PMOP, we compared the correlations of WBC, IL‐6, SII, PCT, and CRP with the lowest BMD T‐score. Significance was denoted by “*” for *p* < 0.05, and “ns” indicated no correlation (Table 2). The results indicated significant correlations between WBC (0.021), IL‐6 (0.044), SII (0.031), and PMOP, while PCT (0.943) and CRP (0.328) showed no significant correlation with PMOP. This conclusion also aligns with the findings of PCA.

**Table 2:** Correlation between inflammatory markers and PMOP.

Inflammatory markers	Correlation coefficient	*p*
WBC	−0.154	0.021(*)
IL‐6	−0.135	0.044(*)
SII	−0.142	0.034(*)
PCT	0.005	0.943(ns)
CRP	−0.066	0.328(ns)

Abbreviations: CRP, C‐reactive protein; IL‐6, Interleukin‐6; PCT, procalcitonin; PMOP, postmenopausal osteoporosis; SII, systemic immune‐inflammation index; WBC, white blood cell count.

### 4.4 Comparison of between‐group differences

**Figure 3:**
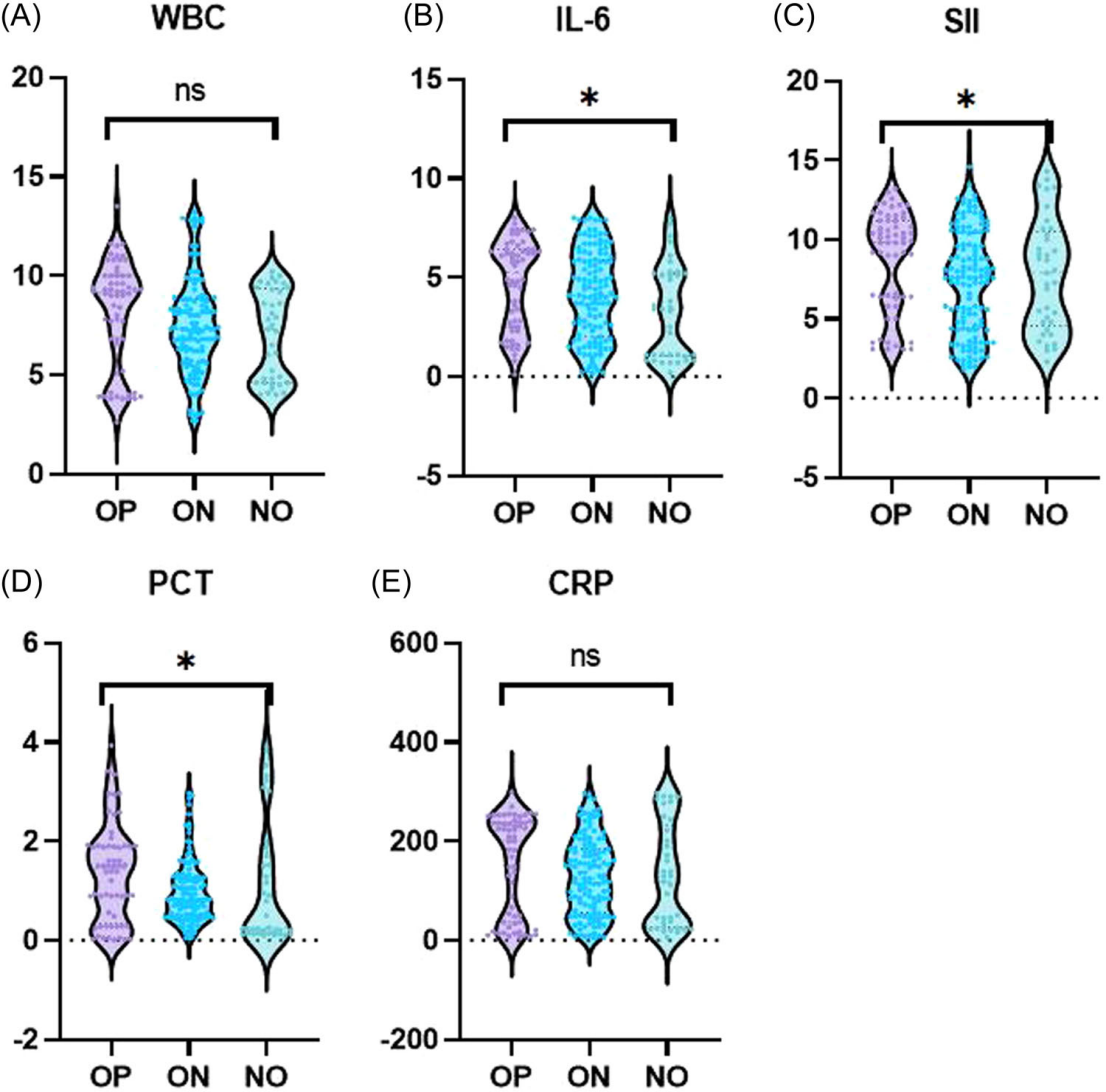
Differential trends of inflammatory markers across varying bone mineral density (BMD) levels. CRP, C‐reactive protein; IL‐6, Interleukin‐6; NO, normal; ON, osteopenia; OP, osteoporosis; PCT, procalcitonin; SII, systemic immune‐inflammation index; WBC, white blood cell count.

PCA and correlation analyses have shown a significant association between certain inflammatory markers in routine blood tests and PMOP. Subsequently, we utilized one‐way ANOVA to analyze the differential trends of these markers across the three groups, further exploring whether they vary with changes in bone mass. The results revealed differential trends for IL‐6 (*p* = 0.0179 < 0.05), SII (*p* = 0.0210 < 0.05), and PCT (*p* = 0.0200 < 0.05) among the three groups. Additionally, no significant differences were observed in WBC (*p* = 0.0526 > 0.05) and CRP (*p* = 0.2035 > 0.05) among the three groups (Figure 3).

### 4.5 ROC curve

We used ROC curve analysis to determine the AUCs of WBC, IL‐6, and SII. Typically, an AUC between 0.5 and 0.7 indicates low predictive accuracy, 0.7–0.9 suggests moderate predictive accuracy, and above 0.9 indicates high predictive accuracy. The cutoff value for predicting PMOP in this study was determined by the maximum Youden's index (sensitivity + specificity − 1) on the ROC curve.

**Figure 4:**
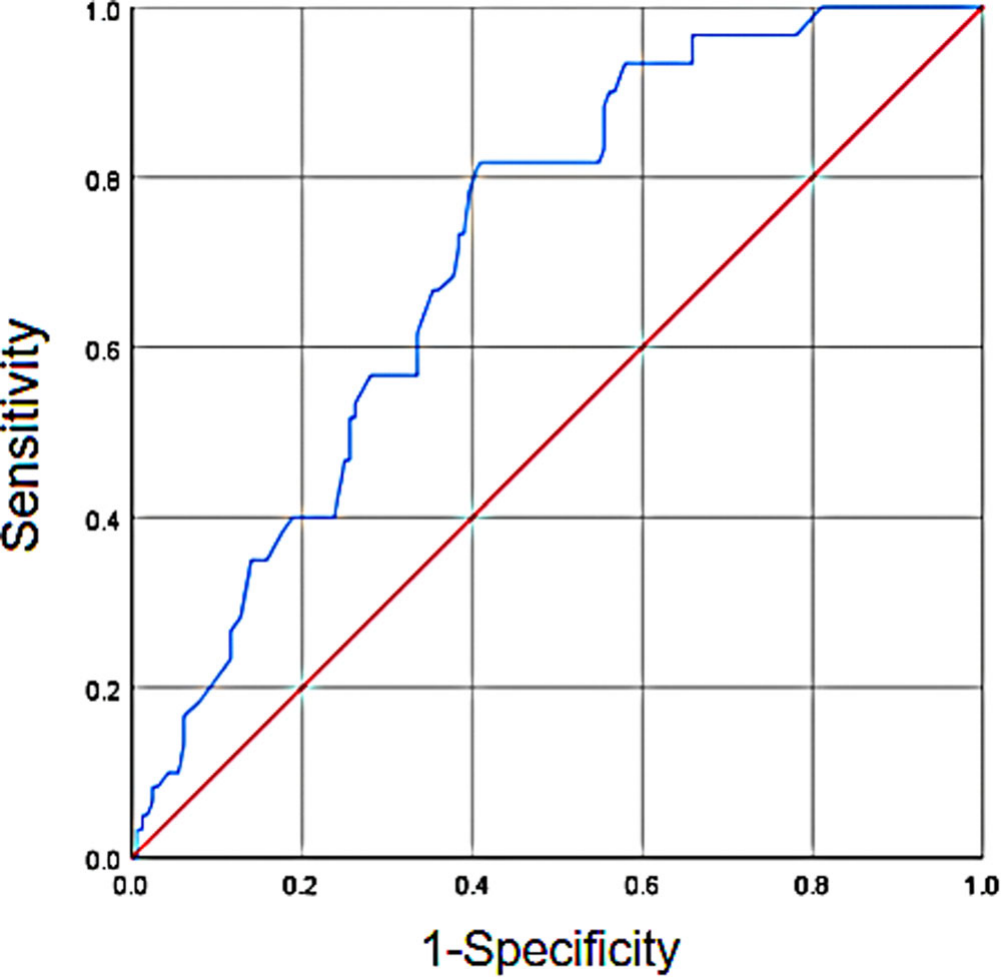
Receiver operating characteristic curve for systemic immune‐inflammation index predicting postmenopausal osteoporosis.

Among the indicators correlated with PMOP mentioned above, SII demonstrates a moderate predictive accuracy (AUC = 0.716), with a maximum Youden's index of 0.408, sensitivity of 0.817, specificity of 0.591, and a cutoff value of 8.05 (Figure 4). Furthermore, SII shows a significant correlation with PMOP (*R* = −0.142, *p* = 0.034 < 0.05), affirming its efficacy as a predictive marker. Conversely, the ROC curves for WBC (AUC = 0.616) and IL‐6 (AUC = 0.598) exhibit AUCs below 0.7, indicating a lower predictive significance.

## 5 DISCUSSION

The primary aim of this study was to investigate the correlation between inflammatory markers detected in peripheral blood and PMOP, analyze their trends, and discuss their predictive value and threshold for PMOP. Results indicate a correlation between inflammatory markers in peripheral blood and PMOP, and their level increases with decreasing BMD. Ultimately, the analysis concludes that the SII holds good predictive value for PMOP.

With the exacerbation of population aging, both the mortality and incidence rates of PMOP have been steadily increasing annually, rendering PMOP a pressing public health concern. Moreover, PMOP contributes to labor force attrition and escalates healthcare expenditures, resulting in economic ramifications. Currently, DXA stands as the gold standard for diagnosing PMOP. However, due to its high cost and limited accessibility, its capability for early screening of PMOP remains inadequate^14^ consequently, numerous scholars are endeavoring to identify more convenient and cost‐effective approaches for preliminary screening and diagnosis of PMOP.^12^, ^15^, ^16^, ^17^ While most studies focus on novel diagnostic technologies or targets, this study explores the early screening value of PMOP through the analysis of classic and readily accessible indicators obtained from routine blood tests.

Numerous studies have confirmed the correlation between OP and immune status. For instance, NF‐κB, a nuclear factor that enhances immunoglobulin κ light chain gene transcription by specifically binding to the κB sequence, is involved in regulating inflammatory responses. Research indicates that inhibiting NF‐κB activation can alleviate skeletal aging and reduce OP.^18^ Reactive Oxygen Species (ROS) are closely associated with inflammation,^19^ and elevated oxidative stress markers in humans have been found to correlate with decreased BMD in postmenopausal women.^20^ Additionally, osteoclasts can promote their generation through immune modulation. For instance, IL‐6 secreted by osteoclasts acts on surrounding osteoprogenitor cells to promote osteoclast formation.^21^ However, clinical research on the relationship between OP and immune status remains limited. Therefore, the clinical phenotypes discovered in this study can provide valuable clinical data for researching the mechanisms underlying the association between immune status and PMOP.

Common inflammatory markers in routine blood tests include WBC, CPR, PCT, IL‐6, among others, and SII can thus be calculated. In this study, we employed PCA to analyze the characteristic values of several inflammatory indicators. The feature values obtained were PC1 = 11.67 and PC2 = 5.662, contributing to a cumulative variance of 72.34% (PC1: 48.71%, PC2: 23.64%). The PCA results indicate that WBC and SII possess significant characteristic values. Furthermore, consistent with two clinical studies, SII was found to be correlated with PMOP.^12^, ^22^ Notably, one clinical study found no significant relationship between WBC and the occurrence or severity of osteoporotic fractures, possibly due to stress‐induced WBC abnormalities resulting from trauma.^23^

Subsequently, we conducted Pearson correlation analyses to examine the relationship between several inflammatory markers and PMOP. The results indicate a correlation between WBC (*R* = −0.154, *p* = 0.021), IL‐6 (*R* = −0.135, *p* = 0.044), SII (*R* = −0.142, *p* = 0.031), and PMOP. Notably, all three correlations are negative, aligning with the notion in the current research context that increased levels of pro‐inflammatory factors and inflammatory markers may lead to PMOP. This clinical perspective further validates these findings.^24^ Using one‐way ANOVA, we analyzed the trends of differences in inflammatory markers among the OP group, ON group, and normal bone mass group. The results reveal significant trends for IL‐6 (*p* = 0.0179 < 0.05), SII (*p* = 0.0210 < 0.05), and PCT (*p* = 0.0200 < 0.05) across the three groups, all increasing with decreasing BMD. This further demonstrates the correlation between elevated inflammatory markers and decreased BMD in postmenopausal women.

To explore the predictive value and threshold of inflammatory markers in peripheral blood routine tests for PMOP, we conducted an ROC curve analysis. The results revealed an AUC of 0.716 for SII, indicating good predictive value, with a threshold of 8.05. Combining these findings, SII exhibits a negative correlation with PMOP. Therefore, when SII exceeds 8.05, it suggests a risk of PMOP. Given the limited clinical research on the threshold for predicting PMOP using SII, these results hold significant clinical implications.

The SII can comprehensively reflect the inflammatory and immune state of the body, as one of the novel inflammatory indexes proposed in recent years.^25^ In a previous study by Lu et al., SII was defined as a new index for evaluating immunity and inflammation, which was obtained by the platelet count × neutrophil count/lymphocyte count. Higher SII indicated that patients had stronger inflammation and weaker immune response.^26^ The conclusion regarding the predictive value of SII for PMOP in this study may potentially be explained by the correlation between neutrophil count, lymphocyte count, platelet count, and PMOP. Studies have shown an increase in neutrophils in ovariectomized mice,^27^ while others have demonstrated high expression of RANKL in neutrophils in patients with OP.^28^ TNF‐α can induce apoptosis of osteoblasts and indirectly stimulate osteoclastogenesis through the Receptor Activator of Nuclear Factor‐κB Ligand (RANKL) produced by B lymphocytes, leading to decreased bone mass.^29^ Additionally, research indicates a correlation between Platelet‐derived growth factor‐BB (PDGF‐BB) and estradiol in PMOP patients.^30^

Several limitations exist in this study. Firstly, this study is a cross‐sectional study, and although an association between SII and PMOP has been demonstrated, a causal relationship between SII and PMOP cannot be inferred. There is a possibility that postmenopausal women may experience inflammation, leading to elevated SII and a subsequent decrease in BMD. Another possibility exists that postmenopausal women may undergo a steep decline in estrogen levels, and numerous studies have indicated a close relationship between estrogen and inflammatory status, that is, estrogen deficiency can enhance inflammatory expression,^31^, ^32^, ^33^ which in turn leads to decreased BMD.^34^

Secondly, The BMD data analyzed in this study did not utilize a fixed BMD measurement at a specific body but rather selected the lowest data from the hip or spine, which somewhat reduces standardization. However, it significantly increases the early screening value for PMOP. This is because the American Association of Clinical Endocrinologists guidelines for PMOP diagnosis recommend using the lowest T‐score from the lumbar spine, femoral neck, total hip, or distal radius.^35^

The sample size in this study was 224 cases, which is inadequate compared to some large‐sample clinical studies. Moreover, the study only included postmenopausal women treated at the Orthopedics Department of Suzhou Benq Medical Center, lacking support of multicenter, multiethnic, and large‐sample data.

Overall, our cross‐sectional analysis results show that there is a potential negative correlation between inflammatory mediators and bone health outcomes in postmenopausal women. Future research needs a large‐scale longitudinal investigation to determine the effectiveness of circulating inflammatory markers as a potential risk factor for OP in menopausal transition period, and the results may provide a theoretical basis for evaluating anti‐inflammatory interventions to prevent the risk of bone loss and fracture in postmenopausal women.

## AUTHOR CONTRIBUTIONS

**Xingyu Jin**: Data curation; formal analysis; investigation; writing—original draft. **Ye Wang**: Writing—original draft. **Huazheng Wang**: Investigation. **Lu Wang**: Writing—review and editing. **Binglong Huan**: Writing—review and editing. **Chao Liu**: Writing—review and editing.

## CONFLICT OF INTEREST STATEMENT

The authors declare no conflicts of interest.

## ETHICS STATEMENT

This study was conducted under the principles outlined in the Helsinki Declaration and its amendments, following the principles of good clinical practice. Approval was obtained from the hospital's ethics committee (Approval No: SZMJYY2022061501), and informed consent was obtained from all patients.

## Data Availability

All data generated or analyzed during this study are included in this published article.
